# Spatiotemporal evolution and spatial benefit analysis of the coupling coordination of China’s three medical systems

**DOI:** 10.3389/fpubh.2026.1782771

**Published:** 2026-04-15

**Authors:** Tian Yue, Zeqiang Zhu, Qianwen Song, Dexun Li, Jing Deng

**Affiliations:** 1 Medical Insurance Office, Shuangliu Maternal and Child Health Hospital, Chengdu, China; 2 School of Integrated Chinese and Western Medicine, Anhui University of Chinese Medicine, Hefei, China; 3 School of Pharmaceutical Economics and Management, Anhui University of Chinese Medicine, Hefei, China; 4 Medical Equipment Department The First People’s Hospital of Shuangliu District, Chengdu, China

**Keywords:** coupling coordination model, healthcare system, medical insurance system, pharmaceutical system, spatial effects

## Abstract

**Objective:**

The harmonious development of healthcare, medical insurance, and pharmaceutical systems is crucial for advancing healthcare reform in China. This study evaluates the coordination level of China’s three medical systems and examines their dynamic evolution trajectories and spatial effects from 2013 to 2023.

**Methods:**

Data from 31 provinces were analyzed using a coupling coordination degree (CCD) model to assess spatiotemporal evolution. Kernel density estimation and Markov chain models were applied to explore dynamic characteristics, while spatial Durbin models were employed to evaluate interregional spillover effects and influencing factors.

**Results:**

From 2013 to 2023, the CCD of China’s three medical systems exhibited a steady upward trend. Eastern regions consistently sustained higher levels of coordination, while central and western regions achieved notable improvements. Some provinces transitioned from disequilibrium to preliminary coordination; Dynamic evolution revealed diminishing absolute disparities but widening relative differences, with intensified multipolarization in eastern and western regions and significant internal variation in central provinces; Spatial analysis identified pronounced spatial autocorrelation and spillover effects, with high-high clustering zones expanding into the Yangtze River Delta, while low-low clustering zones remained concentrated in the northwest. Economic development, urbanization, and human capital contributed significantly to coordination, while government healthcare expenditure exerted a negative impact, and aging effect exhibits a significant threshold effect.

**Conclusion:**

The level of coordination among China’s three major medical systems show sustained improvement, yet significant regional disparities persist, primarily due to uneven pharmaceutical development. Coordination follows a path-dependent trajectory, limiting rapid advancement. Positive spatial spillover effects generate economic influence on neighboring regions. Policies should focus on balancing regional coordination with differentiated strategies, enhancing interdepartmental and interregional mechanisms, and optimizing resource allocation.

## 1 Introduction

The coordinated reform of healthcare, pharmaceuticals, and medical insurance (the “three medical systems”) serves as the core concept for deepening healthcare reform and is recognized as a crucial driver for advancing China’s medical system transformation (1). Since its initial proposal in 2000, central and local governments have issued successive policy documents to bolster the coordinated development of these three systems, establishing it as a central focus in public health policy (2). The level of coordination among healthcare system, pharmaceuticals system, and medical insurance system not only directly affects healthcare service quality improvements but also profoundly influences residents’ health outcomes and the accessibility of medical services, thereby serving as a critical safeguard for achieving universal health coverage (3). However, since the reform and opening-up, China’s healthcare system has transitioned from a government-led public welfare model to a market-oriented, self-financing model, with the profit-driven behavior of medical institutions increasingly evident (4). This reform trajectory has led to uneven resource distribution, with economically developed eastern regions and urban areas possessing greater resources, while central and western regions and primary healthcare settings experience resource constraints. These disparities have further widened differences in healthcare service levels across provinces (5, 6), thereby reducing equity in healthcare services and efficiency in healthcare utilization (7). Additionally, China’s rapid economic growth and accelerating population aging have amplified demand for healthcare services. Nonetheless, persistent challenges—including inefficient pharmaceutical supply chains, imbalanced distribution of medical resources, and mounting pressure on medical insurance funds—have emerged as core bottlenecks hindering healthcare system advancement (8–10).

Against this backdrop, the Sanming healthcare reform model is widely regarded as an exemplary model of “Tripartite Healthcare Reform Coordination” achieving coordinated development of healthcare, pharmaceuticals, and medical insurance through innovative policy design. It has effectively improved healthcare performance, offered valuable insights for nationwide reform initiatives, and evolved into a key focus for national policy (11). Overall, this strategy aims to optimize policies and mechanisms to enhance healthcare efficiency, reduce medical costs, and increase the overall effectiveness of the public healthcare system (12). The coordinated reform of healthcare, pharmaceuticals, and medical insurance represents both a critical approach to addressing current healthcare challenges and an essential component of advancing healthcare system reform. Consequently, effectively integrating resources from these three systems and promoting coordinated reforms has the potential to improve service quality and operational efficiency, thereby laying a solid foundation for realizing the “Healthy China” strategy.

Despite significant disparities in socioeconomic development across China’s regions, less-developed areas experience persistent challenges, including insufficient medical resources, limited medical insurance payment capacity, and underdeveloped pharmaceutical industries (13–16). Consequently, the synergistic effects among healthcare, pharmaceuticals, and medical insurance remain inadequately developed. Advancing coordinated reforms to maximize collaborative efficacy has thus emerged as a central focus for both academic researchers and policymakers. Existing studies on the linkage among healthcare, pharmaceuticals, and medical insurance have evolved from a single-domain focus to multi-system coordination analyses. Early research primarily emphasized efficiency improvements and institutional optimization within individual systems, highlighting localized impacts of policy implementation in healthcare, pharmaceuticals, or medical insurance (17–19). More recently, as the interconnected mechanisms among these systems have been increasingly recognized, research has expanded to examine multi-system coordination. For example, Wang et al. (20) demonstrated that the New Rural Cooperative Medical Scheme significantly increased preventive services and Western medicine usage while reducing reliance on traditional Chinese medicine and lowering patient out-of-pocket expenses. Similarly, Zhou et al. (21) found that medical insurance systems significantly enhanced outpatient and inpatient service utilization among middle-aged and elderly populations, though policy effects varied by insurance type. Wang et al. (22) identified healthcare service capacity development as a critical factor influencing medical insurance fund performance. In addition, scholars have applied coupling coordination models to investigate interactions among the healthcare, medical insurance, and pharmaceutical systems. For instance, Zheng et al. (23) explored the coupling relationship between healthcare and medical insurance coverage, while Hu et al. (24) analyzed the interplay between the pharmaceutical system and healthcare. As research has advanced, the focus has shifted from two-system coupling studies to investigations of three-system dynamics. Guo et al. (25) observed improvements in the overall coupling coordination level of China’s “three-systems linkage” system, although notable regional disparities persist. Song et al. (26) identified spatial clustering characteristics in the coupling relationships among China’s three systems, while Wang et al. (27) highlighted significant intra-regional variations in coupling coordination within China’s central region. Although these studies have significantly contributed to the understanding of China’s coordinated development of the three major healthcare systems, particularly through static analyses of its levels, which allow us to gain an initial understanding of how these levels have developed, this study argues that a dynamic perspective is more crucial. This study contends that a deeper understanding of the coordinated development of China’s three medical systems requires a spatial analysis of their formative mechanisms, future trajectories, and the region-specific impacts on their integration. Consequently, this comprehensive understanding will facilitate the development of targeted policy recommendations for the coordinated development of the three medical systems.

In summary, two significant limitations exist in the current literature regarding the relationship among China’s healthcare, medical insurance, and pharmaceutical system. First, while theoretical coupling relationships between any two systems have been widely studied, a more comprehensive analysis of the dynamic evolution of coupling coordination among all three systems is needed. Second, existing studies largely rely on time-series analyses to assess trends in coupling coordination levels, with few studies adopting dynamic or spatial perspectives to investigate the spatial heterogeneity and effects of coupled coordination development. Consequently, these studies fail to provide more nuanced conclusions.

Based on this, the present paper examines the interconnections and coordinated development patterns among the three healthcare systems across China’s 31 provinces. First, it applies a coupling coordination degree model to investigate the spatial–temporal evolution of coordination among these systems. Second, kernel density models and Markov chain models are employed to explore dynamic evolution trends. Finally, the spatial Durbin model is applied to analyze spatial externalities and the mechanisms influencing their interactions. This study aims to provide decision-making references for provinces and regions to design targeted and effective strategies for the integrated advancement of the three healthcare systems.

The potential marginal contributions of this study are as follows: First, from a theoretical perspective, this paper breaks through the limitations of previous studies that focused solely on pairwise coupling relationships between systems. It centers on the coupling and coordination among the three medical systems, systematically exploring their dynamic evolution patterns and enriching the theoretical framework for their coordinated development. Second, methodologically, this study employs not only traditional coupling coordination degree models to analyze the coordination levels among the three medical systems but also integrates kernel density models and Markov chain models to quantitatively assess the dynamic evolution characteristics of their coupling relationships, revealing their temporal trends. Furthermore, this study employs spatial Durbin models to uncover the spatiotemporal heterogeneity and spatial spillover effects of the coupling coordination relationship from a spatial perspective, addressing gaps in existing literature regarding spatial effects.

## 2 The coupling mechanisms among the three medical systems

### 2.1 The synergistic interaction between the healthcare system and the medical insurance system

The effective coordination between the healthcare system and the medical insurance system serves as the fundamental guarantee for achieving the integrated reform of medical services, medical institutions, and medical insurance (28). As the direct means to realize this integrated reform objective, the healthcare system has significantly enhanced the service efficiency of medical institutions through measures such as establishing a tiered diagnosis and treatment system and advancing medical technologies. This has alleviated the strain on healthcare resources within the healthcare system, reduced healthcare costs, expanded the coverage of medical insurance, and played a positive role in improving the efficiency of medical insurance fund utilization (29). Conversely, the medical insurance system guides the allocation and efficient utilization of healthcare resources through reforms in payment methods. By establishing an actuarial system based on disease economic burden calculations and employing payment models such as Diagnosis-Related Groups (DRG) and Diagnosis-Independent Payment (DIP), the medical insurance system creates effective incentive and constraint mechanisms for the healthcare system. This approach achieves improved resource allocation efficiency, optimized economic operational models, and enhanced cost structures (30).

### 2.2 The synergistic interaction between pharmaceutical systems and healthcare systems

The positive interaction between the pharmaceutical system and the healthcare system is key to enhancing the quality of medical and health services. Through continuous technological innovation and industrial upgrading, the pharmaceutical system provides the material foundation for improving healthcare service quality. Furthermore, the research, development, and application of innovative drugs effectively enhance clinical treatment outcomes. Additionally, the adoption of artificial intelligence diagnostic technologies significantly boosts the service capabilities of medical institutions. The implementation of the generic drug substitution strategy reduces healthcare costs while improving drug supply security at primary healthcare facilities, greatly increasing medical accessibility (31). Conversely, the health demands generated within the healthcare system provide the pharmaceutical system with stable market opportunities and sustained growth momentum, establishing its material foundation for development (32). Moreover, the healthcare system charts the fundamental direction for pharmaceutical advancement by clearly signaling demand for novel drugs, medical devices, and therapies—thus providing the core orientation and impetus for pharmaceutical R&D and innovation (33).

### 2.3 The synergistic interaction between medical insurance and pharmaceutical systems

Policy coordination between the medical insurance system and the pharmaceutical system forms the institutional foundation for ensuring drug accessibility. In practice, China’s medical insurance system has established an effective policy combination through strategic purchasing and dynamic adjustments to the reimbursement catalog, effectively promoting the coordinated development of both systems. On one hand, national negotiations and the “dual-channel” supply system ensure stable drug supply within the healthcare system, alleviate patients’ financial burdens, reduce administrative costs between the two systems, and enhance operational efficiency (34). On the other hand, China’s healthcare system substantially reduces drug prices through negotiations, while extending contractual periods to enhance drug accessibility and sales volume. This approach enhances the stability of profitability for high-quality new drugs, offering some benefit compensation to biopharmaceutical companies developing innovative drugs. It alleviates financial constraints, creates conditions for increased investment in innovation, and effectively promotes innovation and quality improvement within the pharmaceutical system (35).

**Figure 1:**
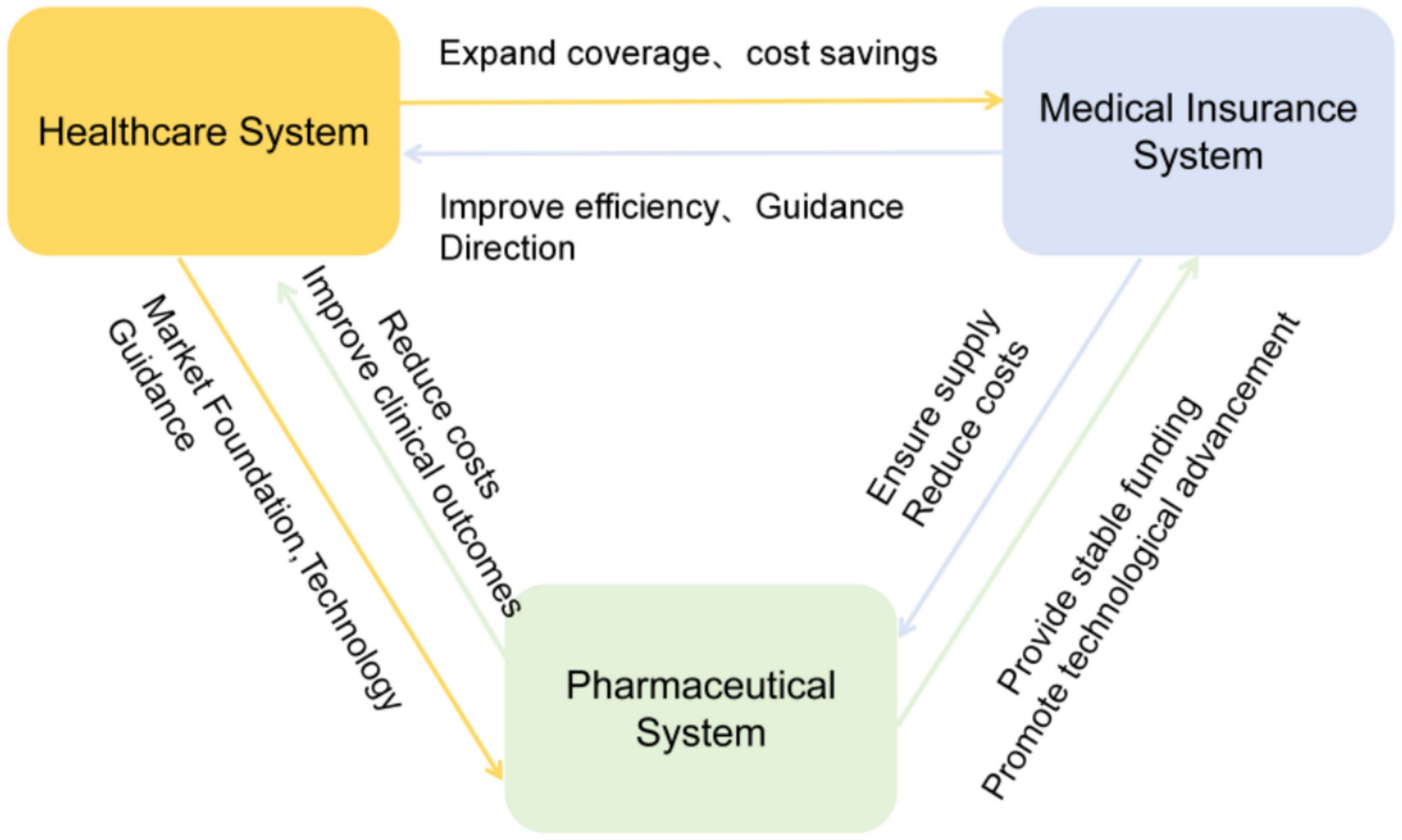
Three-medical-system coordination mechanism diagram.

In summary, the three medical systems are interconnected across multiple dimensions (as shown in Figure 1), including resource allocation, efficiency optimization, coverage levels, industry scale, and innovation capacity. Together, these connections form an organic whole that collectively drives the advancement of healthcare services.

## 3 Data and methods

### 3.1 Indicator selection and data explanation

#### 3.1.1 Construction of indicator system

This paper upholds the principles of scientific rigor, comprehensiveness, representativeness, and practical applicability to develop a CCD evaluation index system that measures the development levels of China’s healthcare system, pharmaceutical system, and medical insurance systems. As outlined in Table 1, the evaluation index for the healthcare system is constructed based on four dimensions—healthcare resources, service scale, service efficiency, and disease prevention and control—by synthesizing insights from existing research (36–38). Medical Insurance System This study draws upon existing research (25, 39–41), focusing on four dimensions: funding levels, benefit levels, coverage rates, and sustainability. Regarding the pharmaceutical system, this study references the findings of Chen et al. (42) and Wang et al. (43) to establish an evaluation framework structured around three dimensions: enterprise scale, economic efficiency, and innovation capacity.

**Table 1:** Evaluation index system of China’s CCD.

First-grade index	Second-grade index	Third-grade index	Weight	Attribute	References
Healthcare system	Health resources	Number of licensed physicians per thousand people	7.60%	+	(36–38)
		Number of registered nurses per thousand people	7.29%	+	(36–38)
		Number of hospital beds per thousand people	11.53%	+	(36–38)
		Number of medical institutions per thousand people	12.09%	+	(36–38)
	Service scale	Number of outpatient visits	9.79%	+	(37, 38)
		Number of hospital discharges	9.67%	+	(37, 38)
	Service efficiency	Hospital bed occupancy rate	11.79%	+	(37, 38)
		Physician workload	10.74%	+	(38)
	Disease prevention and control capacity	Mortality rate for Disease A and B	7.61%	−	(38)
		Incidence rate for Disease A and B	11.90%	−	(38)
Medical insurance system	Funding level	Per capita employee medical insurance funding as a percentage of wages	10.21%	−	(25)
		Per capita urban and rural resident medical insurance funding as a percentage of per capita disposable income	10.69%	−	(25)
		Ratio of basic medical insurance fund revenue to expenditure	6.39%	+	(41)
		Basic medical insurance fund total revenue as a percentage of GDP	9.65%	+	(41)
	Compensation level	Per capita expenditure level of employee basic medical insurance fund	6.27%	+	(39, 41)
		Per capita expenditure level of resident basic medical insurance fund	5.70%	+	(39, 41)
		Reimbursement ratio	8.87%	+	(40)
	Medical insurance coverage rate	Insurance coverage rate	13.98%	+	(40)
		Employee medical insurance participants as a percentage of total insured population	14.15%	+	(39)
	Sustainability	Current fund surplus rate for employee medical insurance	7.60%	+	(39)
		Per capita GDP	6.51%	+	(40)
Pharmaceutical system	Enterprise scale	Number of enterprises	16.70%	+	(42, 43)
		Average number of employees	17.35%	+	(42, 43)
	Economic benefits	Operating revenue	12.99%	+	(42, 43)
		Revenue from new product sales	10.73%	+	(42, 43)
		Internal R&D expenditure	10.13%	+	(42, 43)
	Innovation capability	R&D personnel	8.99%	+	(42, 43)
		Number of valid invention patents	12.91%	+	(42, 43)
		Full-time equivalent R&D personnel	10.19%	+	(42, 43)

### 3.2 Data sources

This study examines data spanning the period from 2013 to 2023, focusing on China’s 31 provinces as the research sample, while excluding the Hong Kong, Macao, and Taiwan regions. To ensure scientific rigor and data comparability, all information is sourced from authoritative publications, including the China Health Statistics Yearbook (2014–2024), China High-Tech Industry Statistics Yearbook (2014–2024), and China Statistical Yearbook (2014–2024), as well as data from the National Bureau of Statistics of China. For regional classification, the study employs the division criteria established during China’s Seventh Five-Year Plan period, categorizing the provinces into three major regions: Eastern, Central, and Western.

### 3.3 Research methods

#### 3.3.1 CRITIC weighting method

The CRITIC weighting method is a technique for comprehensively determining indicator weights by analyzing the comparative strength and conflict between them. Comparative strength is measured using the standard deviation, where a larger standard deviation indicates greater data variation and volatility, resulting in a higher assigned weight. Conflict is quantified using correlation coefficients, with lower correlation between indicators signifying greater conflict, thus assigning higher weights (44). In comprehensive evaluations involving multiple indicators and subjects, the CRITIC weighting method effectively mitigates the influence of highly correlated indicators, minimizes information redundancy, and enhances the reliability of evaluation results (45). Consequently, this study adopts the CRITIC weighting method to calculate the comprehensive development levels of three systems: healthcare, medical insurance, and the pharmaceutical system. The specific steps in the calculation process are outlined below:

Step 1: Data Standardization


$$X_{\mathrm{ij}}=\frac{x_{\mathrm{ij}}-x_{\min}}{x_{\max}-x_{\min}}$$ (1)

$$X_{\mathrm{ij}}=\frac{x_{\mathrm{ij}-}x_{\max}}{x_{\max}-x_{\min}}$$ (2)

In these Equation 1 is used for processing positive indicators, whereas Equation 2 is applied to process negative indicators. In these equations, $x_{\mathrm{ij}}$ represents the raw value of the j-th indicator for the i-th entity, while $X_{\mathrm{ij}}$ denotes the corresponding standardized value.

Step 2: Calculate the Information Capacity of Indicators


$$\begin{array}{c} I_{j}=\sigma_{j}\;c_{j}=\sqrt{\frac{\sum_{i=1}^{m}{X_{\mathrm{ij}}}^{\prime }-\bar{X_{j}^{\prime }}}{m-1}}\;\sum_{i=1}^{m}(1-r_{\mathrm{ij}}) \end{array}$$ (3)

In Equation 3, $I_{j}$ represents the information-carrying capacity of the j-th item, $\sigma_{j}\;$ denotes the standard deviation, which reflects the comparative strength of the j-th indicator, and $c_{j}$ indicates the correlation coefficient, which measures the conflict between the j-th indicator. Additionally, $r_{\mathrm{ij}}$ is the Pearson correlation coefficient describing the degree of correlation between indicators i and j.

Step 3: Calculate Indicator Weights and Evaluation Index


$$\begin{array}{c} T_{i}=\sum_{j=1}^{n}W_{j}{X_{\mathrm{ij}}}^{'}=\sum_{j=1}^{n}\frac{I_{j}}{\sum_{j=1}^{n}I_{j}}{X_{\mathrm{ij}}}^{'} \end{array}$$ (4)

In Equation 4, $T_{i}$ represents the comprehensive development level index of a specific system for the i-th province, while $W_{j}$ denotes the weight assigned to the j-th indicator.

#### 3.3.2 Coupling coordination degree model

The coupling coordination degree model is used to analyze the extent of interdependence and interaction among multiple subsystems by employing the concept of coupling degree. On this basis, the model integrates the coordination development degree to provide a comprehensive evaluation of the overall system (37). The coupling degree quantifies the intensity of interdependence between subsystems, with values ranging from 0 to 1—higher values indicate stronger mutual dependence, whereas lower values suggest weaker interdependence. However, coupling degree alone cannot adequately capture the level of coordinated development among the subsystems. Therefore, the coupling coordination degree is introduced to evaluate the overall coordination and development of healthcare, medical insurance, and the pharmaceutical system. The CCD ranges from 0 to 1, where higher values indicate better coordination among subsystems, and lower values reflect weaker coordination. Considering the extensive interconnections and mutual influences among healthcare, medical insurance, and the pharmaceutical system, this study adopts the coupling coordination degree model to measure and analyze their interactive relationships comprehensively.

The calculation equation is as follows:


$$\begin{array}{c} C=\frac{3^{\sqrt[{3}]{U_{1}U_{2}U_{3}}}}{U_{1}+U_{2}+U_{3}} \end{array}$$ (5)

$$\begin{array}{c} T=\alpha U_{1}+\beta U_{2}+\lambda U_{3} \end{array}$$ (6)

$$\begin{array}{c} D=\sqrt{C\times T} \end{array}$$ (7)

In Equations 5–7, In this context, C represents the coupling degree, D denotes the coupling coordination degree, and $U_{1}$ , $U_{2}$ , and $U_{3}$ correspond to the comprehensive evaluation scores for healthcare, medical insurance, and the pharmaceutical system, respectively. T signifies the comprehensive coordination index for these three subsystems, while α , β and λ are undetermined coefficients. This study assumes that the three subsystems—healthcare, medical insurance, and the pharmaceutical system—are equally important, assigning α = β = λ =1/3. Furthermore, the coupling coordination degree of these three subsystems is classified into ten distinct levels (46), as detailed in Table 2.

**Table 2:** The classification of coupling coordination.

D value range	Harmony level
0 ≤ D < 0.10	Extreme imbalance
0.10 ≤ D < 0.20	Severe imbalance
0.20 ≤ D < 0.30	Moderate imbalance
0.30 ≤ D < 0.40	Mild imbalance
0.40 ≤ D < 0.50	Borderline imbalance
0.50 ≤ D < 0.60	Barely coordinated
0.60 ≤ D < 0.70	Basic coordination
0.70 ≤ D < 0.80	Intermediate coordination
0.80 ≤ D < 0.90	Good coordination
0.90 ≤ D < 1.00	Excellent coordination

#### 3.3.3 Nuclear density estimation

Kernel density estimation is a nonparametric statistical method used to estimate the probability density function of continuous random variables within a given interval. It is widely applied in spatial non-equilibrium analysis, as it allows for the observation of the dynamic evolution of data distribution characteristics—such as shape, location, and polarization—over time through smooth, continuous density curves (47). To effectively capture the dynamic evolution patterns of China’s CCD distribution, this study utilizes the Gaussian kernel density estimation method for measurement.


$$\begin{array}{c} f(x)=\frac{1}{N_{h}}\sum_{i=1}^{N}k(\frac{X_{i}-x}{h}) \end{array}$$ (8)

In Equation 8, *N* denotes the number of observations, *h* represents the bandwidth, $k(\frac{X_{i}-x}{h})$ is the kernel function, $X_{i}$ are independent and identically distributed observations, and *x* is the mean.

#### 3.3.4 Markov chain

Markov chains are a statistical tool used to measure the probability of state transitions over time (48). Spatial Markov chains build upon traditional Markov chains by incorporating geographical spatial influences, enabling analysis not only of the state of a single region but also of its interactions with neighboring areas. This study adopts a quantile-based approach to classify coupling coordination into K distinct types. To examine the dynamic evolution of coupling coordination, an *N × N* Markov transition matrix is constructed, which integrates spatial lag concepts to account for the interactions between the coupling coordination levels of adjacent regions. The corresponding equation equation is provided below:


$$\begin{array}{c} \mathrm{Lag}=\sum Y_{i}W_{\mathrm{ij}} \end{array}$$ (9)

In Equation 9: *Lag* represents the spatial lag quantity, used to evaluate the coupling coordination degree between adjacent regions, $Y_{i}$ denotes the coupling coordination degree of province *i,* $W_{\mathrm{ij}}$ indicates the spatial weight matrix.

#### 3.3.5 Spatial effect analysis method

Spatial autocorrelation analysis is commonly employed to examine whether variables distributed within the same region exhibit mutual dependencies, and it is also used to assess whether variables display spatial clustering characteristics (49). This study utilizes both the global Moran’s I and local Moran’s I indices to test for spatial correlation and spatial clustering in the CCD.

#### 3.3.6 Global Moran’s index

The global Moran’s I index is a statistical measure used to evaluate the spatial distribution characteristics between variables based on their significance levels. A positive significance value indicates a spatial concentration or clustering pattern among the variables, whereas a negative significance value reflects a spatial dispersion pattern. Moreover, the magnitude of the global Moran’s I index reflects the strength of spatial association: values closer to 1 signify a stronger concentration, while values closer to −1 indicate greater dispersion (50). Specifically:


$$\begin{array}{c} \text{Mora}n^{’}s\;\;I=\frac{\sum_{i=1}^{n}\sum_{j=1}^{n}(X_{i}-\bar{X})(X_{j}-\bar{X})}{S^{2}\sum_{i=1}^{n}\sum_{j=1}^{n}W_{\mathrm{ij}}} \end{array}$$ (10)

In Equation 10, $X_{i}$ represents the observed value for region *i*, $X_{j}$ represents the observed value for region *j*, and $W_{\mathrm{ij}}$ denotes the spatial weighting matrix. The Moran’s I index ranges from −1 to 1. A value of *I* > 0 indicates positive spatial correlation, signifying significant spatial clustering of CCD. The closer I approaches 1, the stronger the global positive spatial autocorrelation, and vice versa. Furthermore, when *I* = 0, it indicates the absence of spatial effects.

#### 3.3.7 Local Moran’s index

To compensate for the limitation that the global Moran’s I index cannot precisely identify the specific spatial locations where clustering or anomalies occur, the local Moran’s I index is employed to analyze the local spatial autocorrelation patterns of the CCD. Its specific equation is as follows:


$$\begin{array}{c} I_{i}=\frac{\left(D_{i}-\bar{D}\right)}{S^{2}}\sum_{j=1}^{n}W_{\mathrm{ij}}*(D_{j}-\bar{D}) \end{array}$$ (11)

In Equation 11, when $I_{i}$ > 0, it indicates that the surrounding interval exhibits clustering characteristics, meaning CCD values in the vicinity are relatively close and concentrated, typically manifesting as high-high clustering or low-low clustering. When *I*_i_< 0, it indicates that the surrounding interval exhibits a dispersed state, typically manifesting as high-low clustering or low-high clustering.

#### 3.3.8 Spatial Durbin model

To investigate whether CCD exhibits a spatial spillover effect, this paper constructs a Spatial Durbin Model (SDM) for analysis. The model design is as follows:


$$\begin{array}{c} D=\alpha_{0}+\rho_{1}\mathrm{WD}+\alpha_{1}{\text{controls}}_{\mathrm{it}}+\alpha_{2}{\text{Wcontrols}}_{\mathrm{it}}+\mu_{i}+\lambda_{i}+\xi_{\mathrm{it}} \end{array}$$ (12)

In Equation 12, WD represents the spatial lag term for health capital, capturing the influence of neighboring regions’ coupling coordination degrees on the current region’s CCD. In this study, the spatial weight matrix is based on the spatial distance matrix. Similarly, $\text{Wcontrol}s_{\mathrm{it}}$ denotes the spatial lag term for the series’ control variables, while $\rho_{1}$ and $\alpha_{1}$ represent the spatial lag coefficients for CCD and the control variables, respectively. For the selection of control variables, this study builds on existing research (51–53), incorporating economic, social, and demographic factors such as per capita GDP, urbanization rate (Ubr), and health expenditure as a percentage of GDP (GHI). These variables are used to account for the impact of regional economic development levels and disparities in public resource allocation on CCD. Additionally, educational human capital (Edu) and the old-age dependency ratio (Old) are included as supplementary control variables to enhance the model’s explanatory power. Detailed descriptions of each variable are provided in Table 3.

**Table 3:** Descriptive statistics of variables.

Types	Variable	Number	Mean	SDV	Max	Min
Core variable	Healthcare system	341	0.457	0.067	0.650	0.317
	Pharmaceutical system	341	0.180	0.176	0.883	0.001
	Medical insurance system	341	0.422	0.092	0.733	0.225
	CCD	341	0.527	0.148	0.895	0.215
Control variables	GDP	341	65,900	34,200	216,700	22,400
	GHI	341	8.22%	1.59%	13.93%	3.97%
	Urb	341	60.95%	12.26%	89.58%	23.97%
	Edu	341	2.18%	0.61%	4.37%	0.89%
	Old	341	16.57	4.89	30.6	7.01

**Figure 2:**
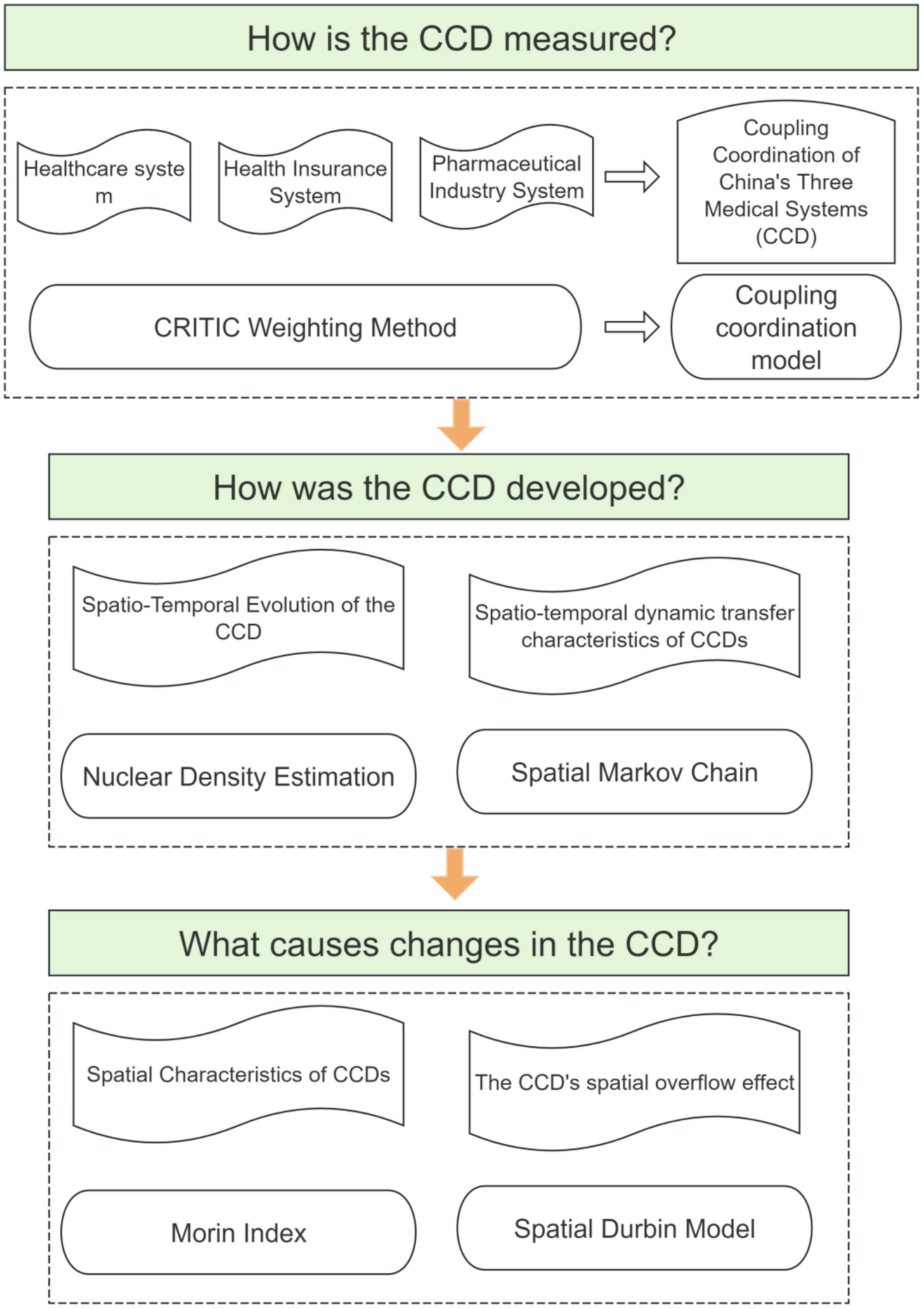
Introduction to method flowchart.

For details on the use of various methods, see Figure 2.

## 4 Result

### 4.1 Time series analysis

**Figure 3:**
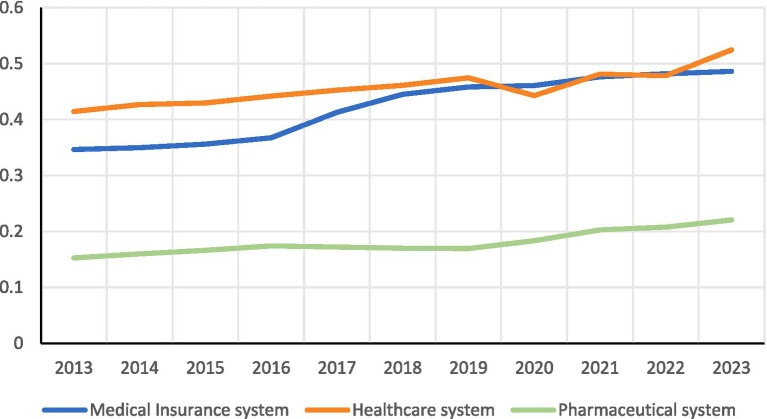
Trends in the development of China’s healthcare, medical insurance, and pharmaceutical system.

As illustrated in Figure 3, China’s healthcare system, pharmaceutical system, and medical insurance system experienced sustained growth between 2013 and 2023. Healthcare system increased from 0.414 to 0.524, reflecting an average annual growth rate of approximately 2.3%. The pharmaceutical system standards saw a rise from 0.153 to 0.221, achieving the highest average annual growth rate among the three systems at 3.7%. Meanwhile, medical insurance system grew from 0.346 to 0.486, with an average annual growth rate of approximately 3.4%. Overall, the levels of medical insurance and healthcare are relatively close. Despite the rapid growth of the pharmaceutical system, its comparatively low initial base has resulted in a significant gap between this system and the healthcare and medical insurance systems. This disparity highlights the pharmaceutical system as a notable weak link in China’s healthcare service system, impeding the coordinated development of the three medical systems.

### 4.2 Spatial distribution characteristics of the three systems

**Figure 4:**
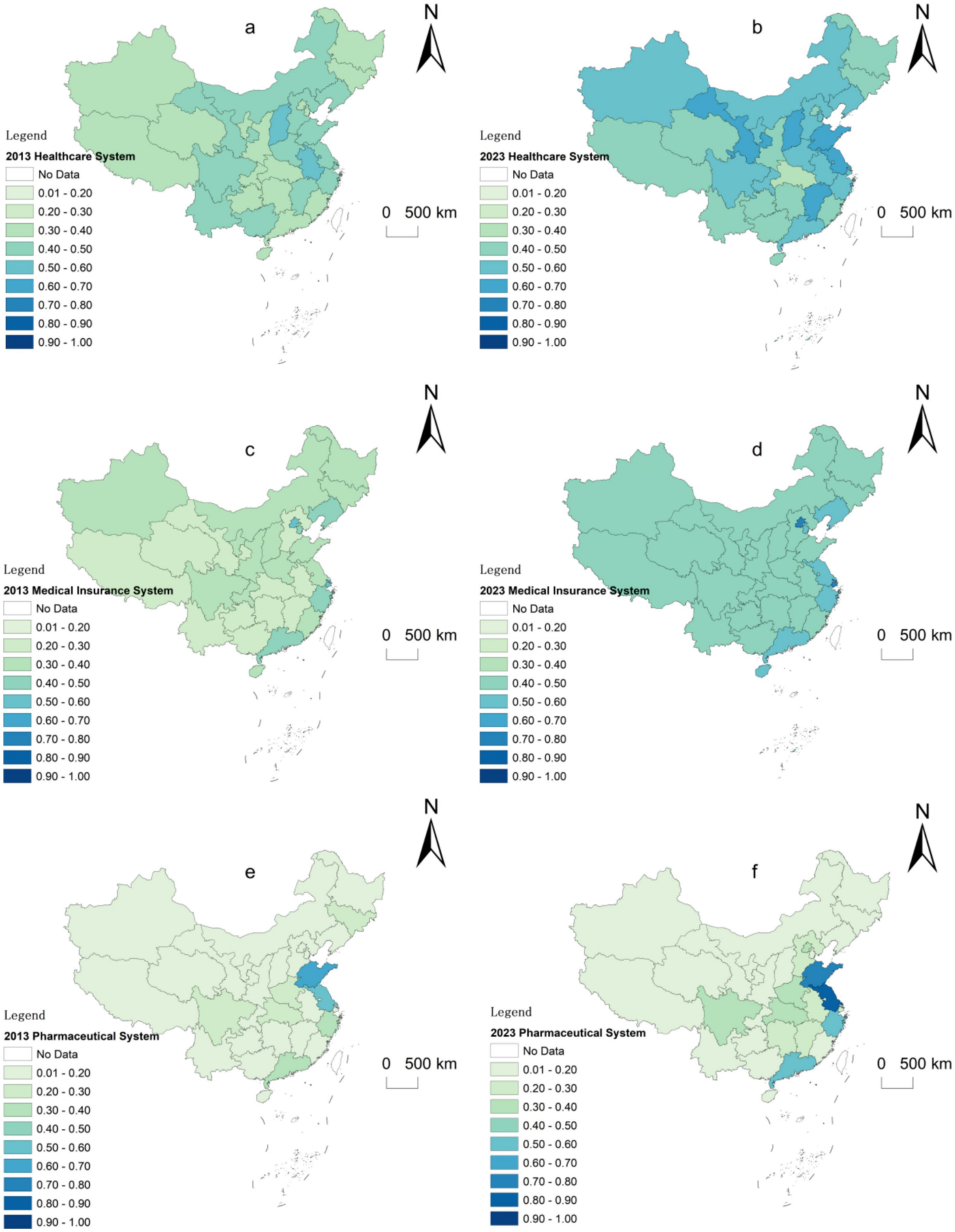
Spatial distribution characteristics of the three medical subsystems. **(a)** Healthcare System Index in 2013, **(b)** Healthcare System Index in 2023, **(c)** medical insurance system index in 2013, **(d)** medical insurance system index in 2013, **(e)** The pharmaceutical system index in 2013, **(f)** The pharmaceutical system index in 2013.

Figures 4a,b illustrate that China’s comprehensive healthcare system evaluation index demonstrated a significant upward trend between 2013 and 2023. Regionally, the eastern provinces maintained a notable lead over the central and western regions; however, this advantage diminished by 2023. The healthcare service capabilities in the central and western regions experienced substantial development during this period, reducing the absolute gap between regions. As a result, China’s spatial distribution of healthcare services shifted from a pattern of high concentration toward a more balanced development across regions.

Figures 4c,d depict a pronounced upward trajectory in China’s comprehensive pharmaceutical system system evaluation index during the same period. In 2013, the eastern region significantly outperformed the central and western regions in pharmaceutical system coverage. By 2023, however, provinces with lower medical insurance levels in the central and western regions achieved significant progress, narrowing the regional disparities. This progress contributed to a more balanced spatial distribution of medical insurance coverage. Nevertheless, economically developed regions, such as Beijing and Shanghai, further strengthened their lead in medical insurance levels. This was partly due to their advanced medical insurance systems, higher levels of digitalization, and superior implementation efficiency relative to other provinces. Additionally, their economic advantages attracted large numbers of migrant workers, whose employment contributions to local medical insurance systems further boosted these regions’ medical insurance capacities.

Figures 4e,f reveal that China’s pharmaceutical system composite evaluation index showed a steady upward trend from 2013 to 2023, while regional disparities remained relatively stable. Leading regions continued to consolidate their industrial advantages, with industrial clustering remaining highly concentrated in areas such as the Yangtze River Delta and Pearl River Delta. In contrast, the central and western regions remained at lower levels of pharmaceutical system development. However, within these regions, distinct patterns of industrial clustering have emerged, with pharmaceutical activity concentrated in traditional strongholds such as Sichuan, Henan, and Hubei. Overall, China’s pharmaceutical system exhibits strong path dependence and persistent regional development imbalances. This suggests that growth in this sector continues to be heavily influenced by locational advantages and established industrial foundations, making it unlikely that interregional disparities will narrow significantly in the near future.

### 4.3 Measurement of coupling and coordination degree in the three medical system

#### 4.3.1 Three medical systems CCD timing variation analysis

**Figure 5:**
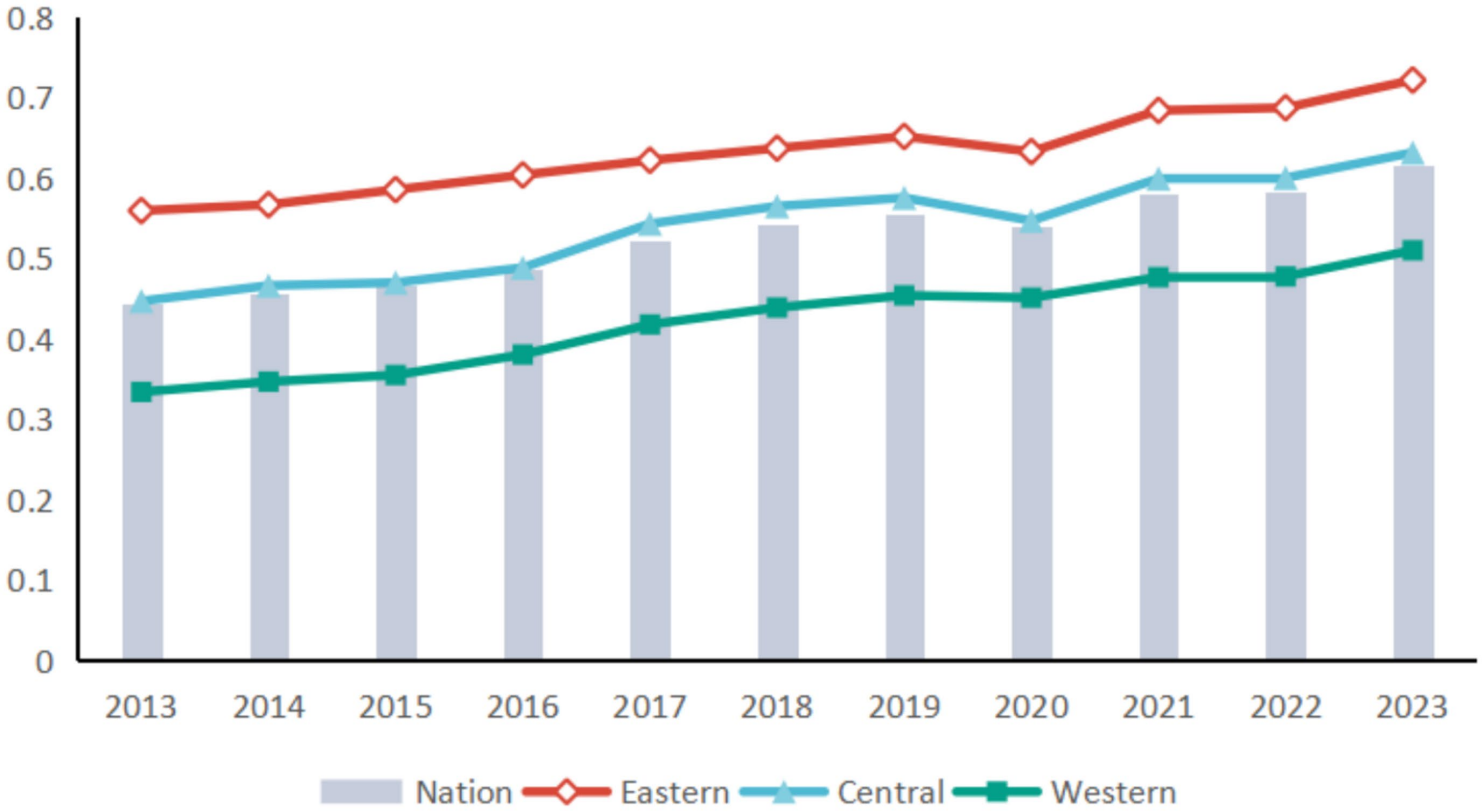
Trends in the development of China’s CCD in the three medical systems.

The coupled coordination model was employed to assess the coordination degree of China’s healthcare system from 2013 to 2023, with aggregated results shown in Figure 5. Overall, the coupling coordination degree rose steadily from 0.443 to 0.616, corresponding to an average annual growth rate of approximately 3.35%. The coordination status improved from mild imbalance to a barely coordinated state. Nevertheless, the three subsystems have yet to achieve high-quality coordinated development. Regionally, pronounced disparities persist: the eastern region maintains a substantial lead, followed by the central and western regions. The central region slightly exceeded the national average, with its coupling coordination index increasing from 0.447 in 2013 to 0.631 in 2023—an average annual growth rate of about 3.51%—marking a transition from near imbalance to the initial stage of coordination. In contrast, the western region continues to lag significantly, remaining well below both the eastern and central regions and the national average.

#### 4.3.2 Analysis of coordination level changes in China’s three medical system

**Figure 6:**
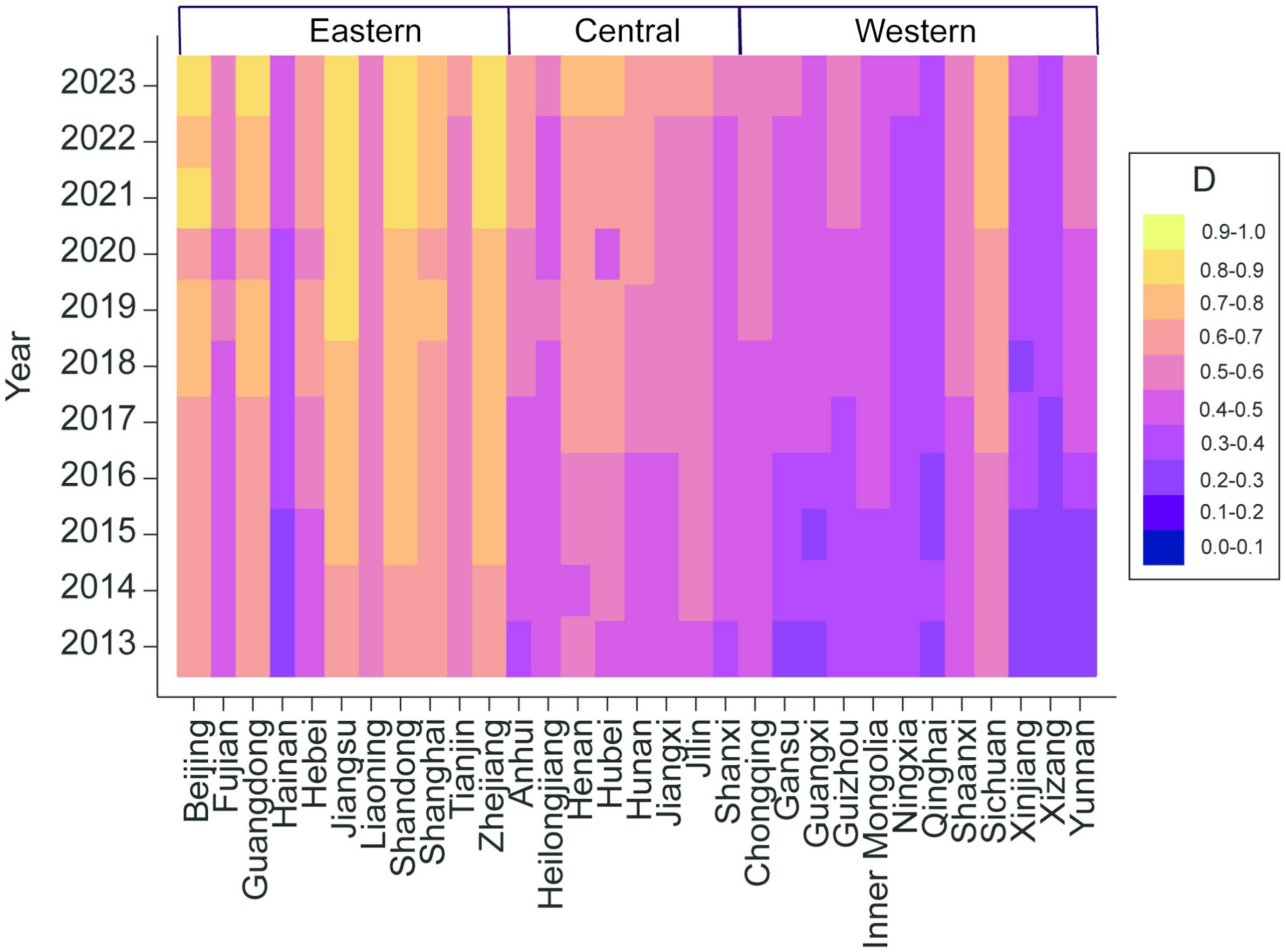
Trends in coordination levels across China’s three medical system.

Due to differences in initial conditions and growth rates across three medical system, the levels and trajectories of coupling coordination exhibit marked heterogeneity across regions and provincial-level administrative units. Overall, regional coupling coordination levels rank from highest to lowest as follows: eastern, central, and western. At the provincial level (Figure 6), the 31 units can be grouped into four categories—Leading, Advantageous, Catching Up, and Lagging. Leading provinces are concentrated in economically advanced eastern regions such as Beijing, Shanghai, Jiangsu, and Guangdong; by 2023, they had transitioned from initial coordination to a well-coordinated phase, representing the highest level of coordination among the three subsystems. Advantageous provinces are primarily located in the central and western regions, including Sichuan, Hubei, and Henan. Leveraging economic, locational, and industrial strengths relative to neighboring provinces, these regions achieved coordination levels substantially ahead of their peers and, by 2023, progressed from barely coordinated to moderately coordinated. The Catching Up category encompasses the developmental status of most provinces—approximately 50%—which trail leading provinces in economic, industrial, and locational factors; by 2023, they had moved from mild imbalance to a barely coordinated state. The Lagging category mainly includes economically disadvantaged and remote western provinces such as Xinjiang, Tibet, and Guangxi; by 2023, they had progressed from moderate imbalance to near imbalance. Although their coupling coordination improved to some extent, these provinces remain in an imbalanced state.

#### 4.3.3 Three medical system CCD kernel density analysis

**Figure 7:**
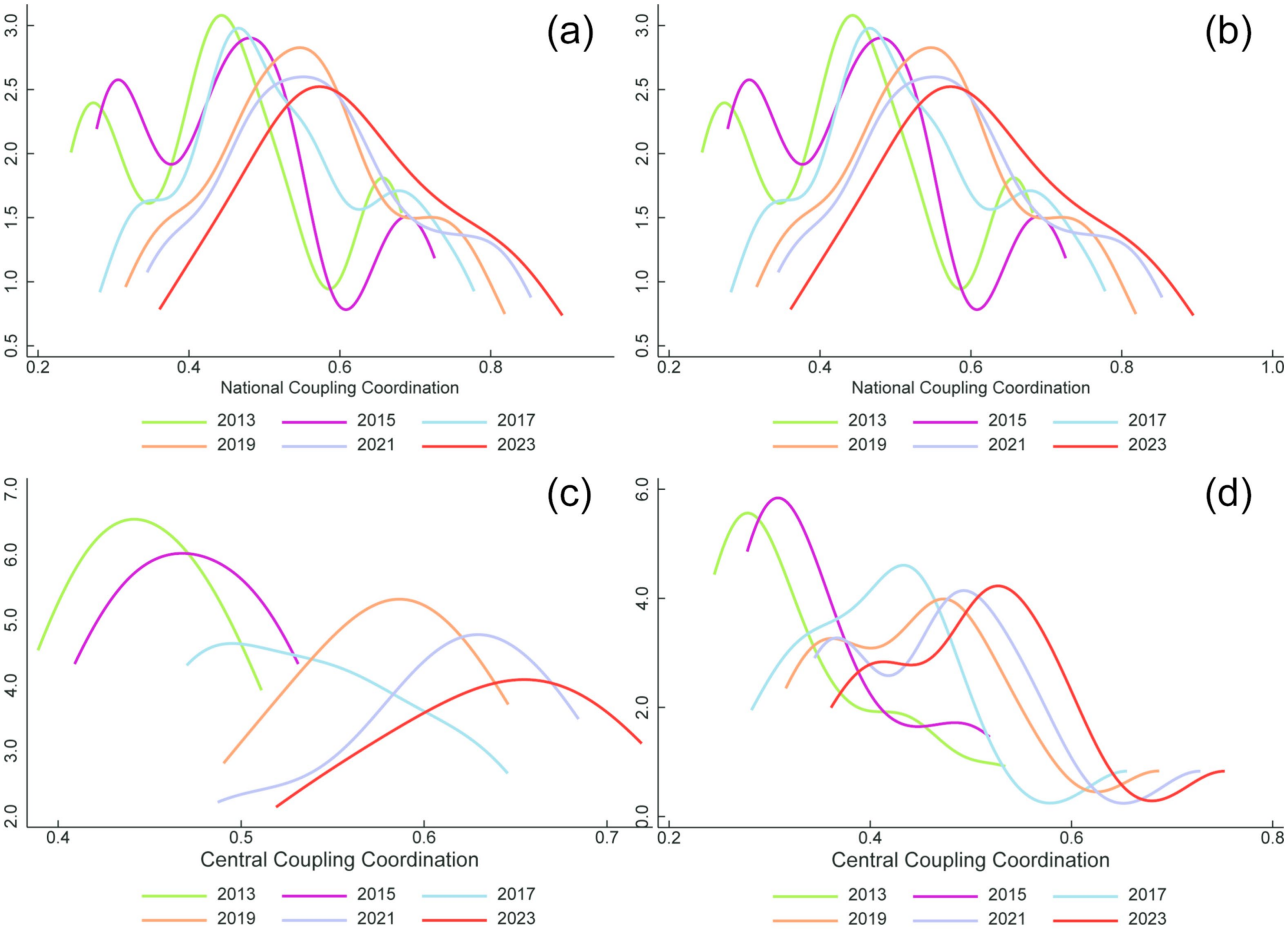
Kernel density estimation of CCD in China from 2013 to 2023. **(a)** National coupling coordination degree, **(b)** Eastern coupling coordination degree, **(c)** Central coupling coordination degree, **(d)** Western coupling coordination degree.

As shown in Figure 7, kernel density estimation is employed to depict the dynamic evolution of coordinated development, examining spatial distribution, distributional shape, and polarization at both national and regional scales. Nationally, from 2013 to 2023, the kernel density curve shifts markedly rightward, accompanied by a decline in peak height and an increase in distributional spread. The transition from a multi-peaked to a single-peaked distribution indicates an overall improvement in the Three-medicals linkage’s coordinated development. At the same time, absolute disparities have widened, while diminishing multipolarity suggests fewer distinct clusters of provinces and, consequently, a relative reduction in interregional differences.

At the regional level, all regions exhibit a pronounced rightward shift, lower peaks, and broader distributions, mirroring the national pattern and indicating steady gains in coupling coordination. As coordination improves, absolute intra-regional differences expand. However, in terms of multipolarity, regional dynamics diverge from the national trend. Both the eastern and western regions transition from unimodal to multimodal distributions and then revert to unimodality. This implies a cyclical pattern of polarization—weakening, then strengthening, then weakening again—meaning that relative disparities within these regions first narrowed, then widened, and ultimately narrowed once more.

#### 4.3.4 Markov chain analysis

To examine steady-state tendencies and transition dynamics of provincial Three-medicals system CCDs and to anticipate their internal characteristics and future trajectories, this study applies a traditional (time-homogeneous) Markov chain to annual CCD tiers. Following the quartile principle, CCDs are partitioned into four levels—low, medium-low, medium-high, and high—denoted I, II, III, and IV.

##### 4.3.4.1 Traditional Markov chain analysis

The transition results in Table 3 indicate the following. First, CCD evolution is stable and continuous, making leapfrogging unlikely: diagonal probabilities dominate off-diagonal entries, no province jumped two tiers in adjacent years, and downward reversion is similarly rare. Second, upward mobility is more prevalent than downward movement: upgrade probabilities exceed downgrade probabilities, indicating a general upward drift in CCD. Third, Tier IV provinces exhibit strong “club convergence,” with a 98.59% chance of remaining in Tier IV and only a 1.41% chance of downgrading. Overall, the three-medicals system’s CCD follows a gradual, path-dependent development pattern anchored in its initial conditions, with limited short-term mobility across tiers.

##### 4.3.4.2 Spatial Markov chain analysis

As shown in Table 4, spatial factors are shown to exert a significant influence on the state transition process related to the classification of China’s Three Medical System CCD development, as evidenced by comparisons of the transition probability matrices of traditional Markov chains and spatial Markov chains. As shown in Table 5, China’s coupling coordination state is characterized by distinct spatial synergy dynamics. Regions with a low coupling coordination level (Types I and II) display a significantly higher number of samples classified as Type I during the t-period compared to similar samples in other neighborhood types. In contrast, regions characterized by high coordination (Type IV) contain 28 samples in the Type IV classification state, which is significantly greater than the number of samples in high-classification states observed in other neighboring region types. Second, the development level of China’s Three Medical System CCD is significantly influenced by the development states of neighboring provinces. Regions adjacent to areas with low coupling coordination levels are characterized by a significant downward shift, as indicated by an increase in the self-retaining probability of low-level states. However, regions neighboring areas with high coupling coordination levels experience increased probabilities of upward transitions for medium-level states, while the likelihood of upward transitions for low-level states unexpectedly decreases. Third, neighboring state types are observed to exert varying impacts on low-value regions. Regions adjacent to Type I or Type IV neighbors exhibit lower upward transitions from Type I compared to traditional Markov models, whereas transitions from Type II or Type III neighbors are significantly higher than those observed among other types. This phenomenon is partly attributed to the resistance faced by low-coordination regions when surrounded, which limits their capacity to secure assistance from adjacent areas during upward transitions. In cases where Type IV neighbors are present, the siphoning effect exerted by high-level regions is found to reduce the likelihood of breakthrough transitions. Furthermore, excessive disparities among industries can hinder the effective absorption of spillover effects from highly developed provinces, thereby contributing to a reduction in breakthrough probabilities when high-value regions are adjacent.

**Table 4:** Markov transition probability matrix for CCD in China’s three-tier medical system.

Type	Spatial lag type	t/(t + 1)	I	II	III	IV	*N*
Traditional Markov chain	No lag	I	0.8554	0.1446	0.0000	0.0000	83
		II	0.0000	0.8228	0.1772	0.0000	79
		III	0.0000	0.0000	0.8571	0.1429	77
		IV	0.0000	0.0000	0.0141	0.9859	71
Spatial Markov chain	I	I	0.9412	0.0588	0.0000	0.0000	34
		II	0.0000	0.6667	0.3333	0.0000	3
		III	0.0000	0.0000	1.0000	0.0000	4
		IV	0.0000	0.0000	0.0000	1.0000	2
	II	I	0.8421	0.1579	0.0000	0.0000	38
		II	0.0000	0.8333	0.1667	0.0000	42
		III	0.0000	0.0000	0.8667	0.1333	15
		IV	0.0000	0.0000	0.0000	1.0000	10
	III	I	0.0000	1.0000	0.0000	0.0000	3
		II	0.0000	0.8387	0.1613	0.0000	31
		III	0.0000	0.0000	0.8857	0.1143	35
		IV	0.0000	0.0000	0.0323	0.9677	31
	IV	I	0.8750	0.1250	0.0000	0.0000	8
		II	0.0000	0.6667	0.3333	0.0000	3
		III	0.0000	0.0000	0.7826	0.2174	23
		IV	0.0000	0.0000	0.0000	1.0000	28

**Table 5:** The spatial correlation test results.

Variable	VIF	Type	Value	*p*-value
GDP	3.29	LM-lag test	182.483	0.01
GHI	1.28	Robust LM-lag test	10.37	0.01
Urb	4.24	LM-error test	246.28	0.01
Edu	2.17	Robust LM-error test	74.168	0.01
Old	1.92	LR-lag test	52.95	0.01
Mean VIF	2.58	LR-error test	40.4	0.01
		Wald-lag test	24.95	0.01
		Wald-error test	13.27	0.02
		Hausman test	7.81	0.25

### 4.4 Spatial analysis of China’s three medical system CCD

#### 4.4.1 Global Moran’s index

Table 6 illustrates that the global Moran’s I index for China’s Three Medicals System CCD increased steadily from 0.31 in 2013 to 0.37 in 2023. The *p*-values for all years were significant at the 1% level, indicating that the spatial distribution of the Three Medicals System CCD is non-random and exhibits a strong positive spatial correlation. This positive spatial correlation may be attributed to ongoing advancements in China’s healthcare, medical insurance, and pharmaceutical systems, along with increasing interregional exchanges and cooperation, which have contributed to the reduction of regional disparities. Concurrently, medical insurance frameworks have gradually expanded beyond provincial boundaries. Furthermore, driven by cost-saving measures and the pursuit of economies of scale, China’s pharmaceutical system has increasingly adopted a clustered development model, effectively reinforcing the positive spatial correlation over time.

**Table 6:** Moran’s I for CCD from 2013 to 2023.

Year	I	E(I)	SD(I)	z	*p*-value*
2013	0.31	−0.03	0.12	2.82	0.00
2014	0.31	−0.03	0.12	2.86	0.00
2015	0.34	−0.03	0.12	3.08	0.00
2016	0.32	−0.03	0.12	2.91	0.00
2017	0.30	−0.03	0.12	2.81	0.00
2018	0.35	−0.03	0.12	3.18	0.00
2019	0.34	−0.03	0.12	3.08	0.00
2020	0.33	−0.03	0.12	3.07	0.00
2021	0.35	−0.03	0.12	3.19	0.00
2022	0.38	−0.03	0.12	3.47	0.00
2023	0.37	−0.03	0.12	3.37	0.00

#### 4.4.2 Local Moran’s index

**Figure 8:**
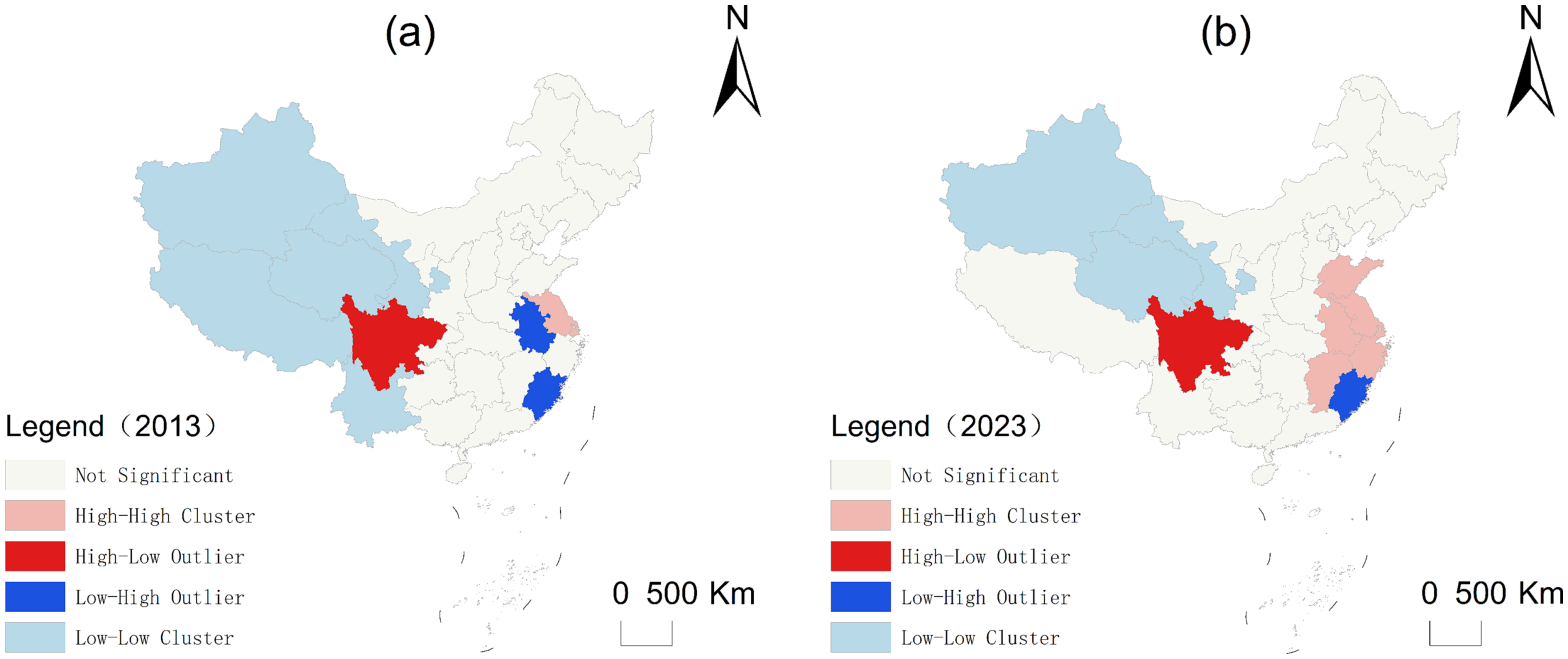
LISA evolution of CCD from 2013 to 2023. **(a)** LISA evolution of CCD from 2013, **(b)** LISA evolution of CCD from 2023.

As shown in Figure 8, the CCD values of China’s Three Medicals System across provinces are predominantly characterized by high-high and low-low clustering patterns. High-high clustering is concentrated in the Yangtze River Delta and adjacent regions, whereas low-low clustering predominantly occurs in western China. Significant temporal shifts were observed in the local spatial correlation characteristics of China’s Three Medicals System CCD during the period 2013–2023. High-high clustering underwent significant spatial expansion, spreading from Shanghai and Jiangsu to encompass the Yangtze River Delta and adjacent regions, with the number of provinces increasing from 2 to 6. Low-low clustering demonstrated a notable contraction, receding from western China to northwestern China and decreasing in extent from 5 provinces to 3. High-low clustering persisted exclusively in Sichuan Province over the 2013–2023 period. Overall, the CCD clustering pattern of China’s Three Medicals System closely aligns with the broader trends observed in China’s economic development. High-value regions have steadily expanded outward from the Yangtze River Delta, whereas low-value regions in western China have gradually contracted, shifting from southwest to northwest.

### 4.5 Spatial model test identification

Analysis of the global Moran’s I and local Moran’s I indices demonstrates the existence of spatial autocorrelation across provinces, autonomous regions, and municipalities. To ensure the robustness of the results, spatial error and spatial lag effects were further analyzed in this study. The variables were subjected to multicollinearity tests prior to conducting spatial panel regression. As shown in Table 5, the mean VIF was 2.58, with a maximum of 4.24, indicating that both values were well below the commonly accepted empirical threshold of 10. This result confirms the absence of multicollinearity issues among the selected variables.

For spatial model selection, this study employs Elhorst’s (54) methodology. As shown in Table 5, LM tests demonstrate that both the SEM and Spatial SAR pass both LM tests and robust LM tests, confirming that spatial effects require spatial econometric modeling. Based on the results of the LR and Wald tests, the SDM significantly rejects regression simplifications to either the SAR or SEM models, at the 5% significance level or better. Results of the Hausman test indicate that the SDM model with random effects is the optimal model specification. Consequently, the SDM model was employed for further analysis, with spatial terms integrated for each variable to establish the model framework.

### 4.6 Analysis of space overflow effects

As shown in Table 7, the *p* value is 0.206 and passes the significance test at the 1% level, indicating that the spatial correlation of the CCD within the Three Medicals System exhibits a significant positive effect. This result suggests that the development of a given region is positively influenced by its neighboring areas. The underlying reason may be attributed to the ability of regions with higher coupling coordination levels to exert a positive “radiating effect” on surrounding lower-value regions through their established systems and resource advantages. This phenomenon is particularly evident in the pharmaceutical system, which demonstrates strong industrial agglomeration effects. The technological, financial, and talent advantages within high-performing regions can drive coordinated industrial growth in neighboring areas, thereby positively affecting the development of the pharmaceutical system in these regions (55).

**Table 7:** The result of Durbin models.

Variable	Coeff	z-value	*p*-value
GDP	0.01	3.62	0.01
GHI	−0.50	−1.91	0.06
Urb	0.89	6.01	0.01
Edu	2.18	1.85	0.06
Old	0.02	1.44	0.15
cons	−0.06	−1.28	0.20
Wx GDP	0.01	1.80	0.07
Wx GHI	−0.17	−0.39	0.70
Wx Urb	−0.22	−1.14	0.25
Wx Edu	2.60	1.53	0.13
Wx Old	−0.01	−3.96	0.01
ρ	0.21	2.79	0.01
R^2^	0.8274		
log-likelihood	666.23		
*N*	341		

In addition, this positive spillover effect is also observed in the healthcare and medical insurance systems. Regions with higher levels of coupling coordination contribute to nearby areas through resource sharing and improved service systems, which promote overall improvements in healthcare service quality and broader medical insurance coverage. The regression analysis provides further insights into the factors influencing the CCD of the Three Medicals System. The regression coefficient for GDP is 0.01 and passes the significance test at the 1% level. This finding indicates that economic growth significantly facilitates the development and coordination of the Three Medicals System. Economic development generates sufficient financial resources for the healthcare, medical insurance, and pharmaceutical system, enabling enhanced service delivery and fostering their coordinated progression (56). The regression coefficient for Urb is 0.888, which is also statistically significant at the 1% level. This demonstrates that urbanization plays a key role in promoting the coordinated development of the three systems. Urbanization creates a large population base that supports healthcare, medical insurance, and pharmaceutical system, allowing them to maximize their service capacity (48). Additionally, urbanization drives significant resource investments, which stimulate industrial development (58), further facilitating the integration and coordination of the Three Medicals System. The regression coefficient for Edu is 2.18 and passes the 10% significance level test, indicating that educational human capital has a substantial positive impact on the CCD of the Three Medicals System. Highly educated professionals contribute to technological innovation in the healthcare and pharmaceutical systems, thereby improving technical capabilities and management practices, which in turn foster coordinated development across the three systems (59). Furthermore, higher educational attainment facilitates access to both material and non-material resources. It is associated with higher income levels, improved employment opportunities, and a greater likelihood of purchasing medical insurance. These factors help alleviate stress on healthcare and medical insurance systems (60), ultimately enhancing the CCD. Conversely, the regression coefficient for GHI is −0.5 and passes the 5% significance level test. This negative coefficient may be due to local government investment preferences in China, which often prioritize short-term, visible-impact projects. Such investments tend to focus disproportionately on expanding medical services while neglecting the pharmaceutical system, leading to imbalances in the development of the Three Medicals System (61). In contrast, the regression coefficient for the proportion of Old is positive, yet it does not pass the significance level test. This outcome may reflect the mixed impact of an aging population: on one hand, the growing older population increases pressure on healthcare, medical insurance, and pharmaceutical systems, hindering their coordinated development (62). On the other hand, the rising demand for healthcare among the older population often drives improvements and upgrades in healthcare systems, enhancing their coordination levels (63). However, significant regional disparities in healthcare service capacity across China may explain the insignificant overall effect. In regions with advanced medical resources, the impact of the older population is relatively minor, while in areas with insufficient healthcare infrastructure, the effect is more pronounced (64).

### 4.7 Further discussion on the impact of old

Based on the findings discussed above, there is a nonlinear relationship between Old and CCD. To explore this further, this study employs a panel threshold regression model to investigate whether this nonlinear relationship exists between Old and CCD. Table 8 presents the bootstrap test results for the impact of Old thresholds on CCD. According to Hansen’s threshold theory (65), in the single-threshold panel model, the F-statistic is 13.93 with a corresponding *p*-value of 0.0033, indicating statistical significance. However, the double-threshold panel model does not pass the significance test at the 1% level. This suggests that the threshold variable for Old passes the single-threshold test with a threshold of 15.06, confirming the nonlinear impact of Old on CCD.

**Table 8:** Threshold test results for the full sample.

Threshold variable	Threshold	Threshold value	RSS	MSE	*F*-stat	Prob	Crit10	Crit5	Crit1
Old	Single	15.06	2.45	0.0079	13.93	0.003	9.34	10.36	12.88
	Double	9.22	2.39	0.0077	6.88	0.1667	7.66	8.78	10.11

As shown in Table 9, when the value of old is below the threshold of 15.06, the regression coefficient is 0.006 and passes the 1% significance level test. This indicates that below the threshold, increased aging promotes the development of CCD. When Old exceeds the threshold of 15.06, its regression coefficient rises to 0.011, which is significantly positive at the 1% level, further amplifying the promotional effect. These results demonstrate that Old has a pronounced nonlinear effect on CCD, consistent with findings from Bo et al. (66) and Wang et al. (67).

**Table 9:** Panel threshold regression results for the full sample.

Variables	Coefficient	Std.	t	P > |t|	[95% conf. interval]	
GDP	0.014	0.0019	7.35	0.000	0.01	0.018
GHI	0.022	0.0029	7.56	0.000	0.015	0.028
Urb	0.003	0.0001	5.75	0.000	0.002	0.005
Edu	−0.021	0.00778	−2.74	0.021	−0.038	−0.004
Old (≤15.06)	0.006	0.0018	3.63	0.005	0.002	0.01
Old (>15.06)	0.011	0.0012	8.93	0.000	0.008	0.0137
_cons	−0.101	0.0396	−2.56	0.028	−0.190	−0.0131

This effect occurs because increasing aging levels heighten residents’ demand for healthcare services and related expenditures, objectively providing the material conditions necessary to sustain and elevate CCD development. However, caution is warranted regarding systemic healthcare risks potentially exacerbated by aging. Rising aging levels may compromise the sustainability of public finances and healthcare security systems (68), intensifying pressures across the three healthcare systems and potentially leading to a decline in CCD.

### 4.8 Reliability testing of the spatial Durbin model

This paper tests the robustness of the benchmark regression model by altering the core explanatory variables and excluding municipalities directly under central government jurisdiction. First, sample estimates are recalculated using CCD, which is replaced by the core explanatory variables after reweighting with entropy weights. As shown in Table 10 (Model 1), even after replacing the core explanatory variables, the spatial autoregressive coefficients remain positive. Additionally, the direction of coefficients and significance levels for most control variables in Model 1 align closely with the original regression results, thereby validating the robustness of the initial findings.

**Table 10:** Robustness test results.

Variable	Model 1	Model 2
GDP	0.01*** (4.51)	0.001 (0.41)
GHI	−0.23 (−1.15)	−0.48** (−2.28)
Urb	0.72*** (6.30)	1.03*** (7.94)
Edu	2.92** (2.90)	1.75** (2.04)
Old	0.48** (2.92)	0.49*** (2.84)
Cons	−0.065 (−0.93)	−0.02 (−0.58)
Wx GDP	0.02** (2.99)	0.01** (2.10)
Wx GHI	0.52 (1.31)	0.07 (0.23)
Wx Urb	−0.22 (−1.00)	−0.74*** (−4.70)
Wx Edu	2.29 (0.90)	0.91 (0.69)
Wx Old	−2.97*** (−4.61)	−0.88*** (−3.60)
ρ	0.39** (3.08)	0.59*** (10.51)
R^2^	0.8985	0.8995
log-likelihood	741.3868	676.5287
*N*	341	297

Next, to account for variations in development levels, resource endowments, and policy support across regions, differences may exist between provinces and cities. Therefore, the four municipalities directly under the central government were excluded for sample regression. As shown in Table 10 (Model 2), after excluding these municipalities, the spatial autoregressive coefficients in the model remained positive, and most control variables exhibited similar patterns. This outcome further confirms the robustness of the original regression results.

## 5 Discussion

Research indicates that since the Chinese government prioritized the coordinated development of healthcare system, pharmaceutical system, and medical insurance system as a key focus for healthcare system reform, the CCD has shown a steady upward trajectory. Notably, after 2016, the level of coordination among these three systems in China advanced rapidly, transitioning from a state of incoordination to one of preliminary coordination. This shift can be largely attributed to the Chinese government’s decision to emphasize healthcare coordination in its government work report for the first time in 2016, thereby elevating it to a national-level policy. This policy prioritization significantly increased the focus of regional governments on integrating healthcare, medical services, and medical insurance, with some regions even incorporating healthcare coordination into government performance evaluation systems. Such measures have further accelerated the process of achieving coordinated development.

Despite these advancements, the study identifies significant industrial gaps and regional disparities within healthcare coordination. Regarding industrial disparities, the pharmaceutical system trails substantially behind the development levels of healthcare and medical insurance. This lag is partly due to the Chinese government’s investment patterns. Some scholars have observed that government investment decisions are often shaped by short-term performance evaluations during officials’ tenure, leading to a preference for projects that yield immediate, visible outcomes (69). In the healthcare system, this trend manifests in the form of prioritizing fixed-asset investments in medical services, leading to rapid expansion in the scale of hospitals and facilities, while contributing to substantial growth in the medical services industry (70). Conversely, the pharmaceutical system, which requires significant, sustained investment and has longer payback periods, faces challenges in securing effective government funding.

Moreover, the pharmaceutical system in China displays notable industrial clustering (71). Due to the sector’s high demands for technology, capital, and research and development, pharmaceutical companies tend to concentrate in economically developed regions with strong locational and resource-based advantages. This pattern has resulted in uneven distribution of resources, technology, and industrial supply chains. Additionally, the implementation of China’s national centralized drug procurement policy may further deepen these disparities. While centralized procurement aims to reduce costs by enabling bulk purchasing (72), it tends to favor larger enterprises with economies of scale. As a result, small and medium-sized pharmaceutical companies in less-developed regions often lose market share, exacerbating developmental imbalances within the pharmaceutical system.

Regarding regional disparities, a tiered distribution pattern is evident across the country, following the order: eastern > central > western regions. At the regional level, economically dominant cities show significant leadership in coordination, consistent with findings by Guo et al. (25). This tiered pattern can be attributed to differences in regional economic strength, resource allocation, and the intensity of policy implementation (73). The eastern region, for instance, has a clear leading advantage in coordinating healthcare, medical services, and medical insurance. This advantage stems from its superior economic and locational conditions, which facilitate the efficient allocation of medical resources, industrial clustering within the pharmaceutical system, and the ongoing optimization of the medical insurance system. Together, these factors accelerate the deep integration of the three systems. In contrast, the central and western regions lag behind their eastern counterparts due to developmental constraints. Despite the national-level strategy for coordinating healthcare, medical services, and medical insurance yielding notable progress in advancing overall integration, disparities in resource endowments, governance capabilities, and developmental foundations have resulted in uneven coordination across regions. These differences have further exacerbated the regional gradient in the coordinated development of healthcare, medical services, and medical insurance.

## 6 Conclusion

(1) From 2013 to 2023, the CCD of China’s Three Medical Systems across its three major regions demonstrated a consistent upward trend. Among these regions, the Eastern Region ranked the highest in CCD, followed by the Central Region, and then the Western Region. The Yangtze River Delta and Pearl River Delta regions continue to lead nationally in coordinated development due to their notable advantages in economic foundations, innovation capabilities, and infrastructure. Moreover, the central and western regions have exhibited faster growth rates, with most provinces transitioning from a state of imbalance to a barely coordinated state.
(2) Regarding its dynamic evolution, the graphical center point of China’s Three Medical Systems CCD displays a noticeable rightward shift, characterized by a reduction in peak height and an increase in peak width. The trend toward multipolarity is gradually diminishing, suggesting that although absolute regional disparities in the coupling coordination of the Three Medical Systems have narrowed, relative differences between regions are gradually increasing. This phenomenon is particularly evident in the eastern and western regions, where multipolarization intensifies significantly, leading to a marked increase in internal regional disparities. By contrast, the central region demonstrates more pronounced absolute disparities between advantaged and disadvantaged provinces. Furthermore, the transfer characteristics of the CCD levels in the Three Medical Systems highlight path dependency effects, which make cross-level transfers less likely. Spatial factors reveal that neighborhood types play a critical role in the dynamic evolution of coordinated development, with strong spatial spillover effects. Provinces bordering high-level regions have a higher probability of upward transfers, benefiting from their proximity to these more developed areas. However, neighboring high-level provinces can also experience siphoning and competitive effects—proximity to multiple high-level provinces may intensify competitive pressures, increasing the risk of downward transfers. Conversely, adjacency to low-level provinces typically triggers siphoning effects, which decrease the likelihood of upward transfers for those low-level provinces.
(3) The CCD of China’s Three Medical Systems exhibits a significant spatial correlation, with an overall upward trend over time. High-high clustering emerges as the dominant development pattern, steadily expanding outward from the Yangtze River Delta to its surrounding areas. Conversely, low-low clustering demonstrates a shrinking trend, becoming more concentrated in Northwestern China.

Additionally, the CCD of China’s Three Medical Systems displays strong spatial spillover characteristics, exerting positive spillover effects on neighboring regions. Key factors contributing to enhanced regional coordination include the level of economic development, the degree of urbanization, and educational human capital. However, government health expenditure has a negative impact on coordination development. Meanwhile, the effect of aging on the coordinated development of the Three Medical Systems remains inconclusive.

## 7 Policy implications

Based on the analysis of the spatiotemporal evolution of the CCD within the Three Medical Systems, the coordinated development of healthcare, medical insurance, and pharmaceuticals across China should adhere to the principle of balancing regional coordination with tailored, localized approaches. Governments should establish cross-departmental and cross-regional policy linkage mechanisms through institutional innovation, effectively harnessing the driving force of regional integration strategies. Such mechanisms can facilitate the efficient flow and optimal allocation of healthcare, medical insurance, and pharmaceutical resources across regions, enabling more balanced development nationwide.

For relatively underdeveloped regions such as Tibet, it is recommended that the central government increase financial support and incorporate the proportion of health expenditures into local government performance evaluations. The focus should be on enhancing funding guarantees for grassroots public health services and improving the service capacity of primary healthcare institutions. Additionally, by leveraging technologies such as artificial intelligence, telemedicine, and smart wearables, collaborative relationships can be established with high-level hospitals to effectively extend the reach of quality medical resources. This approach will prioritize improving access to quality healthcare in remote and underserved areas, thereby addressing resource scarcity challenges.

For central provinces in catch-up mode, such as those leveraging the Yangtze River Economic Belt and the “Central China Rise” strategy, efforts should be made to actively receive industrial transfers from eastern provinces within the pharmaceutical system. These regions should proactively establish local pharmaceutical supply chain systems to promote the sustainable development of the pharmaceutical system. Concurrently, within the healthcare system, attention should be focused on addressing disparities between urban and rural areas, concentrating efforts on enhancing county-level medical service capabilities. Accelerating the construction of regional medical centers will facilitate the expansion and decentralization of high-quality medical resources.

Additionally, provincial-level reforms for pooling medical insurance funds should be expedited to bolster the risk-bearing capacity of social security systems. The implementation of DRG payment systems should also be promoted to reduce transaction costs and foster closer collaboration between healthcare, medical insurance, and pharmaceutical systems.

Furthermore, leveraging platforms such as pharmaceutical system parks can drive the synergistic development of the pharmaceutical supply chain and healthcare service systems. This approach supports the advancement of an innovation-driven, full-chain development model. Building upon these efforts, China should continuously optimize the pathway for coordinated development among healthcare, medical services, and pharmaceuticals (74). This can be achieved through the establishment of a multi-level collaborative governance system, complemented by dynamic monitoring and evaluation mechanisms. Such measures will help reduce regional development disparities, promote health equity, and provide strong support for realizing the Healthy China vision.

## 8 Limitations

This study has several limitations that should be addressed in future research. First, due to constraints in publicly available data, the analysis is limited to the provincial level, omitting municipal and county-level evaluations. As a result, future studies should expand their focus to include municipal and county-level analyses to better capture localized dynamics and variations in the CCD of the Three Medical Systems.

Second, although this study utilized the most recent data available, the reliance on statistical yearbooks introduces inherent data lag, as these publications often reflect information from one or more years earlier. To improve the accuracy and relevance of findings, future research should seek to incorporate more up-to-date data sources.

Third, this study primarily measures the coordination of the “Three Medical Systems” (healthcare, medical insurance, and pharmaceuticals) using macroeconomic, healthcare, and social data. While considerable efforts were made to account for the interconnections across these dimensions, the interactions within the individual systems—such as the deeper dynamics between healthcare, medical insurance, and pharmaceutical supply chains—are difficult to capture comprehensively using macro-level data alone. This limitation may result in certain inaccuracies when analyzing micro-level mechanisms. Therefore, in future research, we will strive to develop an indicator system that more comprehensively reflects China’s three healthcare systems. Particularly within the pharmaceutical system, this should encompass not only aspects related to the pharmaceutical manufacturing industry but also residents’ perceptions of medications and the optimization of interactions between systems.

## Data Availability

The original contributions presented in the study are included in the article/Supplementary material, further inquiries can be directed to the corresponding authors.
